# The measurement of the surface energy of solids using a laboratory drop tower

**DOI:** 10.1038/s41526-017-0031-y

**Published:** 2017-10-12

**Authors:** Alfredo Calvimontes

**Affiliations:** Predevelopment, Product Division Dish Care, BSH Hausgeräte GmbH, Robert-Bosch-Straße 16, 89407 Dillingen an der Donau, Germany

## Abstract

This work presents a technique for the study and measurement of the interfacial energies of solid–liquid–gas systems. The instrument and the evaluation method for the measurements obtained by it, allow the analysis of the energy changes of sessile drops submitted to microgravity. A mathematical model based on the thermodynamic of wetting is applied to evaluate the interfacial energies as a function of the drop shape changes due to the effect of the release of gravitation during the experiment. The presented model bases on the thermodynamic equilibrium of the interfaces and not on the balance of bi-dimensional tensors on the contour line. For this reason, the model does not follow Young’s equation as the current surface wetting characterization techniques usually do.

## Fluids: Microgravity releases the tension

Gravity is shown to influence the shape of a small drop of water on a surface in a theoretical model developed by a researcher in Germany. Alfredo Calvimontes from BSH Hausgeräte GmbH demonstrates the importance of the droplet geometry in determining the interfacial energies. Surface tension strongly influences a water drop’s shape, but it was thought that gravity played little role for very small drops. However, recent experimental work has suggested that this might not be true. Calvimontes develops a theoretical model that differs from the conventional approach of the two hundred- years-old Young’s equation in that it assumes a thermodynamic equilibrium of the interfaces, rather than a balance of forces, on the solid-liquid-gas contour line. The model was supported by high-speed-camera images of droplets on various surfaces in free fall using a three-meter drop tower that allows quantifying the change of shape from normal gravity to microgravity.

## Introduction

Young^1^ proposed two hundred years ago that the contact angle of the three-phase contact line results from the balance of three tensors. This idea can be expressed by the following equation:

1 $$\gamma _{\rm S}\; = \;\gamma _{{\rm SL}}\; + \;\gamma _{\rm L}{\mathrm{cos}}\theta _{\rm Y}$$

where *γ*_S_, *γ*_SL_, and *γ*_L_ represent the interfacial tensions per unit length of the solid–vapor, solid–liquid, and liquid–vapor contact lines respectively, i.e., the surface tensions, and *θ*_Y_ is the contact angle.

In principle, there are three important conditions for applying Young’s equation^2^: the surface has to be chemically homogeneous, completely flat and smooth, and the solid–liquid–vapor system must be free of the effects of gravity. Under these conditions, Eq. (1) represents the mechanical balance of three surface tensions along the contour line of the three phases. This balance has also been derived using the principle of minimizing the total free energy of the system.^3–5^ Most recent thermodynamic derivation relies on interpreting *γ*_S_, *γ*_SL_, and *γ*_L_ as scalar thermodynamic surface energies instead of tension vectors.^4^

According to Makkonen,^6^ a very important reason for adopting the surface energy interpretation is that, while *γ*_SL_ and *γ*_L_ can be interpreted either way, the surface tension on a dry solid, *γ*_S_, is a contentious concept.^3,7–11^ Bikerman^8^ and Ivanov et al.^9^ have argued that Young’s equation is not a balance of forces. At the same time, the surface energy interpretation has led to many misunderstandings of the wetting phenomenon on patterned surfaces.^12,13^ The validity of Young’s equation was questioned^6^ at the nanoscale^14–17^ and on flexible surfaces^17,18^ as well.

According to Leger and Joanny,^19^ the effect of body forces such as gravity on the contact line is small for small drop volumes. Gravity would affect the shapes of wetting liquid drops in their central region where they are flattened, but in a small region, close to the contact line, one would expect the liquid–vapor and liquid–solid interfaces to make an angle given by Young’s law. These observations were supported by the theoretical calculations of Fujii and Nakae.^20^ According to Leger, Joanny, Fujji and Nakae, only forces that become increasingly large at the contact line such as the viscous force on an advancing liquid can affect Young’s law. However, recent experimental evidence using microgravity by parabolic arc flights^21^ and microgravity drop towers^22,23^ have demonstrated that the effect of gravity on the contact angle is relevant even at very small drop volumes such as 5 µL. According to Allen,^24^ who studied the wetting of very small drops with small contact angles, a drop is small enough to neglect gravitational influences only if its volume is less than 1 µL.

Of the four parameters of Young’s equation, only *γ*_L_ and *θ*_Y_ can be readily measured; hence, this equation can only provide the difference between the solid–vapor surface tension *γ*_S_ and the solid–liquid interfacial tension *γ*_SL_. For this reason, an additional equation providing a relation among the surface tensions in Young’s equation is required, which is,

2 $$\gamma _{{\rm SL}} = f\left( {\gamma _{\rm S},\gamma _{\rm L}} \right)$$

Such an equation is referred to as an equation of state for interfacial tensions.^25^ Combining Eq. (1) with Eq. (2), we yield

3 $$\gamma _{\rm L}\cos \theta _{\rm Y} = \gamma _{\rm S} - f\left( {\gamma _{\rm S},\gamma _{\rm L}} \right)$$

This equation was the start point for several attempts to obtain mathematical expressions or numerical procedures able to provide the values of *γ*_S_ and *γ*_SL_ when only the values of *γ*_L_ and *θ*_Y_ are known. The most relevant solutions^26^ were given by Fox and Zisman,^27^ Owens and Wendt,^28^ Janczuk and Bialopiotrowicz,^29^ Wu,^30^ van Oss, Chaudhury and Good,^31^ Li and Neumann,^25^ Kwok et al.,^32^ Shimizu and Demarquette^33^, and Chibowski et al.^34^ Except for the Li-Neumann method, all the mentioned solutions use a pair or more liquids to calculate the surface energy of the solid and the interface solid–liquid. Experimental results of Hejda et al.^24^ show that the solution of the mathematical approaches strongly depends on the liquids used. According to these authors, the approach proposed by Li and Neumann is also impractical because of its strong dependence on the liquids used for the calculations.

For all of the above, it is necessary to revisit the phenomenon of wetting by paying more attention to its surface nature as much as the derived approaches of Young’s equation have been put in the balance on the contact line of the three phases.

## Theoretical Basis of the Method

The major thermodynamic quantity which characterizes a surface or an interface is the reversible work, *γ*, to create a unit area of surface at a constant temperature (*T*), volume (*V*), and chemical potential of component *i* (*µ*_*i*_). This quantity is not equal to the surface free energy except under certain conditions.^35^ The term surface tension was used to describe the contractile nature of surface films, i.e. their tendency to minimize surface area. This term became so entrenched in the literature that it is widely used today. Thus *γ* is widely called the surface tension, though the meaning of those words may have little physical significance in many situations. As mentioned above, the quantity *γ* is the scalar thermodynamic surface energy that characterizes the thermodynamic property of an interface.

The *γ* of a newly created surface is defined as

4 $$\gamma \; = \;\frac{{dw}}{{d\Omega }}$$

i.e., the specific surface works to form *d*Ω, a new surface area.

The thermodynamics of interfaces is the same as the thermodynamics of homogeneous systems except that the work term of conventional thermodynamics must include all the *γ d*Ω components for the heterogeneous (interface-containing) systems.^35^ In systems where charges or electrical potentials are present, the electrical work must also be included. This is usually done by means of the electrochemical potential.

By open systems with surfaces, in the absence of other forms of work, e.g. electrical, magnetic, gravitational, etc., the Internal Energy *E* can be defined as

5 $$dE\; = \;TdS - PdV + \mathop {\sum}\limits_i {\mu _idN_i + \gamma d\Omega }$$

The Enthalpy *H* is

6 $$dH = T\,dS - V\,dP + \mathop {\sum}\limits_i {\mu _i\,dN_i + \gamma \,d\Omega }$$

The Helmholtz energy *A* is

7 $$dA = - S\,dT - PdV + \mathop {\sum}\limits_i {\mu _idN_i + \gamma d\Omega }$$

And the Gibbs free energy *G* is

8 $$dG = - SdT + VdP + \mathop {\sum}\limits_i {\mu _idN_i + \gamma d\Omega }$$

By these systems, the creation of a new area of surface, *d*Ω, may cause a flow of *dN* molecules to or from the surface region, resulting in concentration profiles,^36^ which lead to a surface excess (or deficiency) of component *i*:

9 $$\Gamma _i = \frac{{dN_i}}{{d\Omega }}$$

As a consequence:

10 $$\mathop {\sum}\limits_i {\mu _idN_i = \mathop {\sum}\limits_i {\mu _i} \Gamma _id\Omega }$$

From basic thermodynamics, the chemical potential of component *i*, *µ*_*i*_, is given as

11 $$\mu _i = \left( {\frac{{\partial A}}{{\partial N_i}}} \right)_{T,V,N_j,\Omega }$$

12 $$\mu _i = \left( {\frac{{\partial G}}{{\partial N_i}}} \right)_{T,P,N_j,\Omega }$$

13 $$\mu _i = \left( {\frac{{\partial E}}{{\partial N_i}}} \right)_{S,V,N_j,\Omega }$$

where *P* = pressure, *S* = entropy, *N*_*j*_ = the number of molecules other than type *i*, and Ω = the surface area.

Using Eq. (9) and applying basic thermodynamics, one can develop expressions for surface energy, surface Gibbs free energy and surface Helmholtz energy

14 $$\left( {dE} \right)_V = TdS + \gamma d\Omega + \mathop {\sum}\limits_i {\mu _id\Gamma _id\Omega }$$

15 $$\left( {dA} \right)_{T,V} = \gamma d\Omega + \mathop {\sum}\limits_i {\mu _id\Gamma _id\Omega }$$

16 $$\left( {dG} \right)_{T,P} = \gamma d\Omega + \mathop {\sum}\limits_i {\mu _id\Gamma _id\Omega }$$

The corresponding specific energy and specific free energies are

17 $$\left( {\frac{{dE}}{{d\Omega }}} \right)_V = e_s = T\frac{{dS}}{{d\Omega }} + \gamma + {\sum} {\mu _i\Gamma _i = specific\,surface\,energy}$$

18 $$\left( {\frac{{dA}}{{d\Omega }}} \right)_{T,V} = a_s = \gamma + {\sum} {\mu _i\Gamma _i = specific\,surface\,Helmholtz\,energy}$$

19 $$\left( {\frac{{dG}}{{d\Omega }}} \right)_{T,P} = g_s = \gamma + {\sum} {\mu _i\Gamma _i = specific\,surface\,Gibbs\,free\,energy}$$

At constant *T*, *P*, *S*, *V*, and if Γ_*i*_ = 0, *γ* = *e*_*s*_ = *a*_*s*_ = *g*_*s*_, but only under these conditions.

In general, the surface Helmholtz and Gibbs free energies and the surface “internal” energy are different quantities. Because pressure is a more constant parameter than volume in heterogeneous systems, Helmholtz energy is usually preferred over Gibbs free energy.^35^

The process of forming a new surface can be divided into two parts:

1. The phase must be cleaved to expose the new surface
2. Atoms in the surface plane rearrange to assume their equilibrium positions

In a multicomponent system, part 2 may also be combined with the migration of bulk atoms to or from the interface, i.e., the development of surface excesses or deficiencies, Γ_*i*_. In a liquid, parts 1 and 2 occur nearly instantaneously. In a solid, part 2 may occur very slowly or not at all.^35^

In a one-component system, Γ_*i*_ = 0 unless there is such a stoichiometric restructuring around the interface so as to significantly change the density of the phase near the surface.

Therefore, at a constant density in a single-component system *γ* = *a*_*s*_ = *g*_*s*_, or *γ* is both the specific Gibbs free energy and the specific Helmholtz energy. Thus, *γ* can be called the specific surface free energy. At constant *S* (no morphological restructuring), *γ* will also be the specific surface energy.

*γ* is a thermodynamic property of an interface; it is not in general *g*_*s*_, *a*_*s*_, or *e*_*s*_, though it may be equal to one, two, or all three of these quantities under certain conditions. The specific surface work of Eq. (4) is commonly called the “surface tension”. For this reason, “surface tension” and “surface free energy” are synonymous with the understanding that both really are the specific surface Helmholtz energy.

On the other hand, surface stress is the work necessary to stretch or compress an existing surface. In a liquid this cannot be done without causing more atoms to join the surface; i.e., creating a new surface. Hence in liquids, the surface stress is *γ*_L_ (the reversible work to form new surface, i.e., surface tension. In a solid, this is not so because the lack of mobility of atoms means that a process can stretch them without causing bulk atoms to join the surface planes. Thus, the process may stretch or compress the surface of a solid without changing the number of atoms in the surface, but only their distances of separation, thus producing a surface stress.^35^

**Fig. 1:**
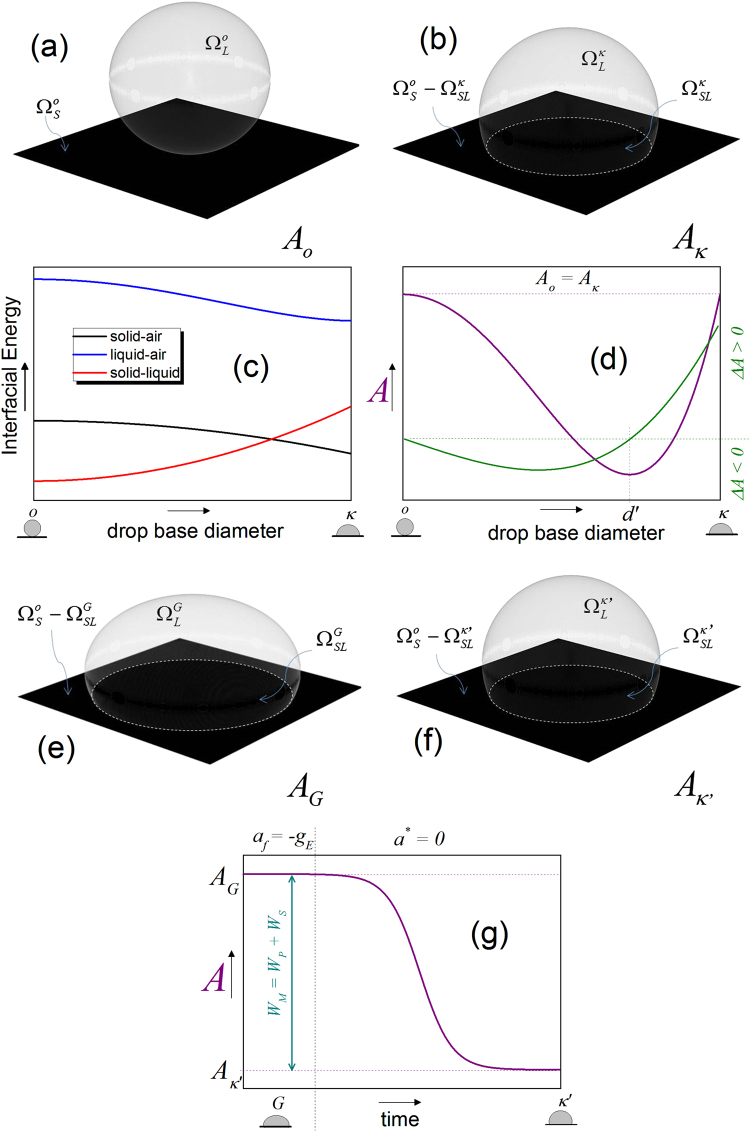
Thermodynamics of wetting without gravity and after “switching off” gravity. **a**, **b** In the absence of gravity, the drop of the state A_o_ **a** spontaneously spreads on the surface and reaches the state *A*_*κ*_ **b**. **c**, **d** Schematic representation of the interfacial energy changes **c** and the total Helmholtz energy changes **d** of the system while a drop spontaneously wets a solid surface without the effect of gravity. **e**, **f** By the free fall, the energy that the terrestrial gravitational field is producing on the droplet **e** is released, letting the droplet reach a new equilibrium only governed by the interfacial energies **f**, which shape is a spherical dome. **g** The free fall makes the system release the energy with which the gravitational field was deforming the drop. The Helmholtz energy of the drop in the form of interfacial energies is released, resulting in a drop with a spherical dome shape, the configuration *κ*′

Let’s consider a system where, in the initial condition that we will call the configuration *o*, the lower end of a liquid drop is at an infinitesimal distance from a smooth solid flat surface of area $\Omega _S^{\mathrm{o}}$ in a gas medium (Fig. 1a). No force field is affecting the system (the acceleration of the system is zero, i.e. a* = *0*). Due to the absence of gravity, the shape of the drop is a perfect sphere of area $\Omega _L^{\mathrm{o}}$ = *4π* $r_{\mathrm{o}}^{\mathrm{2}}$, where *r*_*o*_ is the radius of the sphere. Under these conditions, and only due to the work developed by the surface energies of the system, the drop spontaneously spreads the surface (Fig. 1b) up to reach the configuration *κ*. This results in the formation of $\Omega _{SL}^\kappa$, an interfacial area between the solid and the liquid. The area of a perfect spherical dome formed by the interface liquid–gas is $\Omega _L^\kappa$.

A quantity of importance in this analysis, as demonstrated above, is the change—the difference of Helmholtz energy between each two contiguous *j-*configurations of the system during the spontaneous wetting process.^37^ The term “configuration” refers to a state in which the drop is at rest in a position of metastable equilibrium. Associated with each configuration are the characteristic interfacial areas $\Omega _{SL}^j$, $\Omega _L^j$ and a characteristic Helmholtz energy, *A*_*j*_.

For a given configuration, according to Eq. (18), and at constant *T*, *V*, and if Γ_*i*_ = 0 (one-component liquid, solid and gas)

20 $$\Delta A_j = \begin{array}{*{20}{c}} {\Delta \sum \left( {\gamma \,\Omega } \right)_j} \\ {{\mathrm{all}}\,{\mathrm{interfaces}}} \end{array} - \begin{array}{*{20}{c}} {\Delta \sum \left( {\gamma \,\Omega } \right)_i} \\ {{\mathrm{all}}\,{\mathrm{interfaces}}} \end{array}$$

Let’s take as an example a system in which *γ*_L_ > *γ*_SL_ > *γ*_S_. During the spontaneous wetting of the droplet of Fig. 1a, b, the Helmholtz energy of the system decreases because part of the liquid–gas interface creates a new solid–liquid interface (Fig. 1c). The energy of the solid–gas interface contributes additionally to the creation of the solid–liquid interface. The wetting process ends when the system reaches the equilibrium in the configuration *κ*.

During the first part of the process, from the configuration *o* to *d*′ is fulfilled the condition *ΔA*_*j*_ < 0 (Fig. 1d). One part of the energy of the system is momentarily stored inside the liquid due to the morphological restructuration of the molecules that increases the internal energy of the drop, i.e., *A*_*o*_ − *A*_*d*′_ *=* *TΔS* *=* *ΔE*, see Eq. (17).

The wetting process could end precisely at the configuration *d*′, where the Helmholtz energy of two contiguous *j-*configurations presents no more change (*ΔAj* = 0). However, the internal energy stored inside the drop during the wetting between *o* and *d*′ can make the work necessary to continue wetting from *d*′ up to the equilibrium configuration *κ*, i.e., *ΔE* = *A*_*κ*_ − *A*_*d*′_. At this point, the configuration *κ*, the Helmholtz energy of the system recovers the value of the initial configuration *o*. In other words, comparing the configurations *o* and *κ*, no work has been done on the system, nor has it done any work on its surroundings. The net change of Helmholtz energy during the wetting process is zero.

21 $$\Delta A_{o\, \to \kappa } = A_\kappa - A_o = 0$$

Equation (20) represents the ideal wetting in the absence of gravity. In a real process without gravity, part of the available surface energy will be lost in the form of entropy during wetting and *A*_*k*_ will be slightly smaller than *A*_*o*_. This fact will ensure that the wetting continues from *d*′ to *κ* and not go back to the initial configuration *o*.

Continuing with the ideal model of spontaneous wetting, from Eq. (20)

22 $$\Delta A_{o \to \kappa } = \gamma _L\Omega _L^\kappa + \gamma _{SL}\Omega _{SL}^\kappa + \gamma _S\left( {\Omega _S^o - \Omega _{SL}^\kappa } \right) - \gamma _L\Omega _L^o - \gamma _S\Omega _S^o = 0$$

That results in

23 $$\gamma _L\left( {\Omega _L^\kappa - \Omega _L^o} \right) + \left( {\gamma _{SL} - \gamma _S} \right)\Omega _{SL}^\kappa = 0$$

A parameter *κ* can be now defined as

24 $$\kappa = \frac{{\Omega _L^o - \Omega _L^\kappa }}{{\Omega _{SL}^\kappa }}$$

*κ* is a dimensionless parameter that characterizes a liquid drop at equilibrium, resting on a flat surface without the effect of gravity. Under the condition of weightlessness, this parameter is, in principle, independent of the size of the drop and not equivalent to the cosine of the contact angle of Young’s model.

25 $$\gamma _{SL} - \gamma _S = \kappa \gamma _L$$

In Young’s model, *cosθ*_Y_ represents the bi-dimensional fraction of the liquid surface tension acting horizontally on the triple contact point solid–liquid–gas. By contrast, in the present model, *κ* represents the ratio of the liquid–gas interface area-decrease and the solid–liquid interface area created by wetting (Eq. (24)). While *cosθ*_*Y*_ applies only to the boundary line of the bi-dimensional drop profile, *κ* applies to all the interfaces of the three-dimensional system.

Let’s consider now, as initial condition, configuration *G*, a drop resting in equilibrium on a solid and flat surface under the effects of the terrestrial gravitational field (*a*_1_ = *−g*_*E*_, *g*_*E*_ = 9.81 m/s^2^), see Fig. 1e. Suppose that the system is inside a closed capsule that is submitted to the free fall under controlled conditions.

If *a*_2_ is the acceleration of the uniformly accelerated motion of the capsule downwards, in the case of the free fall, its value is obviously *−g*_*E*_. This will switch off the effects of the gravitational field inside the capsule that contains the surface and the drop. The resulting acceleration of this system will be given by

26 $$a^* = - a_2 + a_1$$

So, in the case of free fall, *a* ^*^ = 0, that corresponds to an inertial system inside the capsule. To reach this condition, the energy with which the terrestrial gravitational field is flattening the drop must be released, letting the drop reach a new equilibrium state (Fig. 1f) only governed by the interfacial energies—the configuration *κ*′. Macroscopically, it will result in the receding of the drop by the decrease of the interfacial area and its “deformation” to a perfect sphere due to the increase of its height. The acceleration that modifies the drop shape during the very short time of the mentioned energy release process is given by *a*_*f*_:

27 $$a_f = - a_2$$

During the energy release process, the receding of the drop will result in contact angle hysteresis. The multiplicity of apparent contact angles, which is an essential feature of contact angle hysteresis, is associated with the multiplicity of equilibrium states that a drop may assume on a rough or heterogeneous surface.^38^ One apparent contact angle is associated with the stable equilibrium state (the global minimum in the free energy of the system). The others are linked to metastable equilibrium states (local minima in the free energy). The transition between metastable states, toward the stable equilibrium state, depends on the energy available to the drop for overcoming the energy barriers which inherently exist between the metastable states.^38^

By definition, the phenomenon of hysteresis is observed when a parameter of the system, such as the volume of a drop placed on a solid, is varied back and forth, or when an external force is making the drop move in one direction (ie. by the tilting table experiment). In the present study, the release of gravitational energy makes the contour of the drop recede on the surface and experience hysteresis. For this reason, the present model is, in principle, oriented to characterize chemically homogeneous and smooth surfaces.

According to Grundke,^39^ while the advancing contact angle is expected to represent the property of the predominant material of the surface, the receding contact angle is only a manifestation of the impurities of that solid surface. It has been shown,^38^ that the contact angles assumed by the drop during advancing or receding is oscillatory. These oscillations may explain the macroscopic stick-slip behavior which is observed during contact angle hysteresis measurements. The present model does not take into account the contact angle values, because they only provide information about balance on the drop line contour. However, the hysteresis effect occurs during the drop recedes and it can modify the increase–decrease of the interfacial areas during de-wetting. The model evaluates the shape of the droplet in the configuration *κ*′, when the oscillations are already macroscopically concluded. In this configuration, under microgravity, the drop shape should be a perfect spherical dome. However, depending on the surface inhomogeneities of real materials, and on the fact that microgravity does not mean the absolute absence of gravity, the configuration *κ*′ could also be a metastable state on which the effects of hysteresis are not at all negligible. This topic will be studied in more detail using the experimental results below.

Going back to the case of the sessile drop in the free fall, the acceleration experienced by the drop for the short time of the energy release is *a*_*f*_ *=* *+* *g*_*E*_, i.e., a short duration force acting upwards. The mechanical work that the drop must make to modify its shape and release surface energy is given by *W*_*M*_.

28 $$- W_M = \Delta A = A_{\kappa {\prime}}\; - A_G$$

From the standpoint of the drop, this work can also be divided into two components: the work *W*_*P*_ necessary to move up its center of mass and the work *W*_*S*_ necessary to radially move the contour line solid–liquid–gas during a short de-wetting process (Fig. 1g).

29 $$W_M = W_P + W_S$$

The change in the potential energy of the droplet can be calculated by measuring the change in the position of all the liquid particles, by considering for a moment the liquid as a particle system. If a particle system is changing from the state 1 to the state 2, their potential energies under a force field that produces the acceleration *a*_*f*_, can be described by

30 $$e_{p1} = \mathop {\sum}\limits_i^n {m_ia_fz_{1i}}$$

31 $$e_{p2} = \mathop {\sum}\limits_i^n {m_ia_fz_{2i}}$$

where *m*_*i*_ is the mass of each particle and *n* the total number of particles. The change in potential energy from state 1 to state 2 is given by

32 $$W_p = a_f\left( {\mathop {\sum}\limits_i^n {m_iz_{2i}} - \mathop {\sum}\limits_i^n {m_iz_{1i}} } \right)$$

The center of mass of a particle system is defined by

33 $$z_c = \frac{{\mathop {\sum}\limits_i^n {m_iz_i} }}{m}$$

Then, Eq. (32) for the potential energy necessary to move all the liquid particles from the state G to *κ*′, can be written as

34 $$W_p = ma_f\left( {z_{c\kappa {\prime}} - z_{cG}} \right) = \rho Va_f\left( {z_{c\kappa {\prime}} - z_{cG}} \right)$$

where *ρ* is the density of the liquid and *V* is the drop volume.

**Fig. 2:**
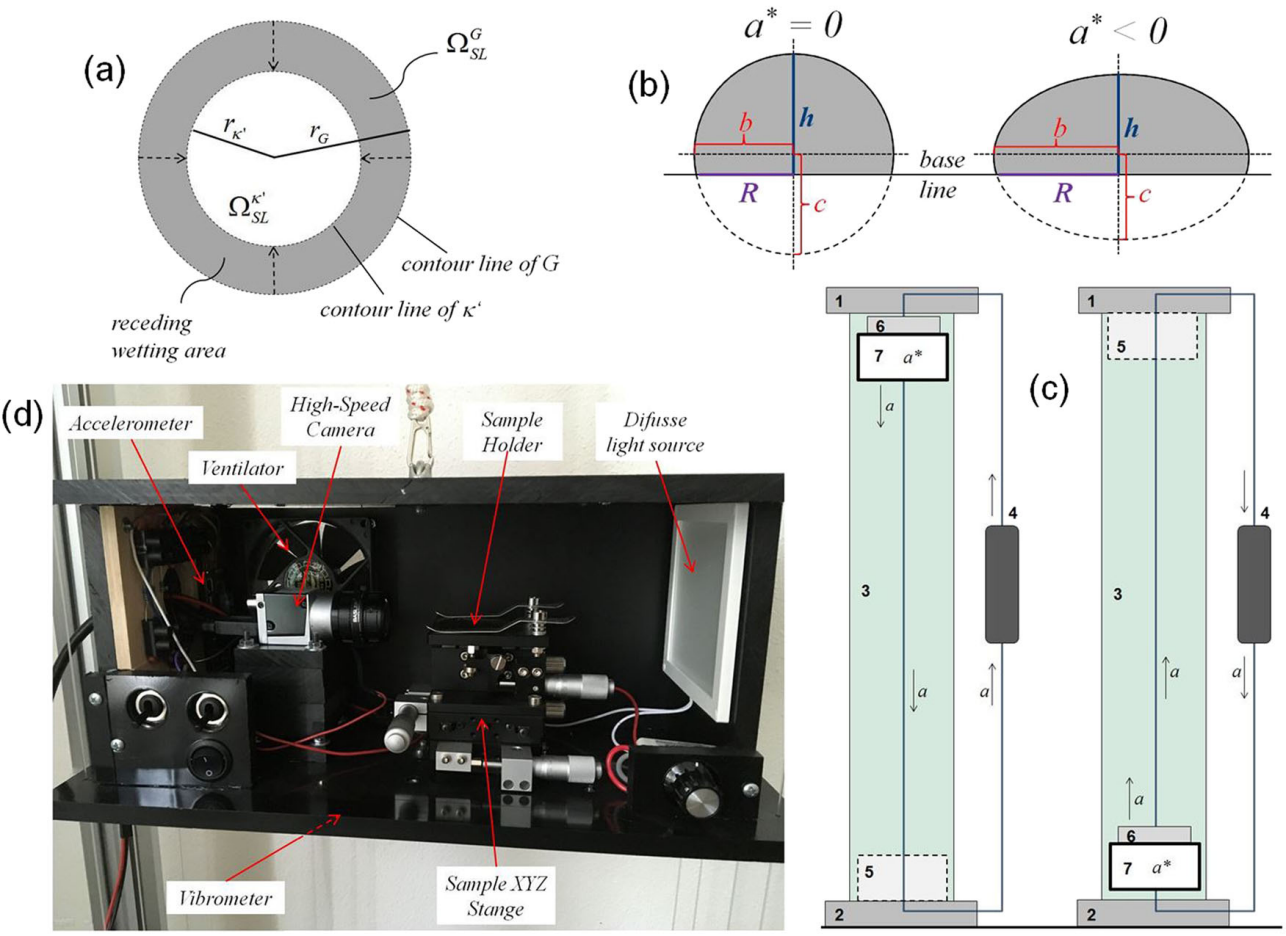
Deweting, drop profiles and the laboratory drop tower. **a** The release of the force field leads to de-wetting. A receding work decreases the interfacial area from $\Omega _{SL}^G$ to $\Omega _{SL}^{\kappa {\prime}}$. **b** Profiles of the two possible spheroids of the drop shape: sphere and oblate. The 2D- profiles of the spheroid caps are in gray. In the profiles of this example, the contact angle is larger than 90 degrees. The same concept can be applied in the case of angles below 90 degrees. **c** Laboratory acceleration tower: the measure capsule (7) can be moved upwards or downwards. **d** Measuring capsule used for the experiment contains a high-speed camera, XYZ-stage, light diffusion source, accelerometer and vibrometer, and cooling system

The second component of the work that the drop must make, the energy necessary to produce the receding wetting during its deformation from the state *G* to *κ*′, can be calculated by the integration of all the differentials of energy needed to move the contour line on the surface in the radial direction (Fig. 2a).

35 $$\mathop {\int}\limits_G^{\kappa {\prime}} {dW_s} = \mathop {\int}\limits_G^{\kappa {\prime}} {\tau ldr}$$

where *τ* is tension necessary for the drop to move the contour line on the surface, *l* is the length of the contour line, and *r* the radius of the wetting area Ω_SL_. These considerations result in

36 $$W_s = 2\pi \tau \mathop {\int}\limits_G^{\kappa {\prime}} {rdr}$$

which, in turn, results in

37 $$W_s = \tau \pi \left( {r_{\kappa {\prime}}^2 - r_G^2} \right) = \tau \left( {\Omega _{SL}^{\kappa {\prime}} - \Omega _{SL}^G} \right)$$

At the equilibrium, the work made by the drop—the energy released—will result in energy changes on the system surfaces. Equations (28), (29), (34) and (37) result in

38 $$- \rho Va_f \left( {z_{c\kappa {\prime}} - z_{cG}} \right) - \tau \left( {\Omega _{SL}^{\kappa {\prime}} - \Omega _{SL}^G} \right) = \gamma _L\left( {\Omega _L^{\kappa {\prime}} - \Omega _L^G} \right) \\ + \gamma _{SL}\left( {\Omega _{SL}^{\kappa {\prime}} - \Omega _{SL}^G} \right) + \gamma _S\left( {\Omega _S^o - \Omega _{SL}^{\kappa {\prime}} - \Omega _S^o + \Omega _{SL}^G} \right)$$

Making the corresponding arrangements, we get

39 $$\left( {\gamma _{SL} - \gamma _S + \tau } \right)\left( {\Omega _{SL}^{\kappa {\prime}} - \Omega _{SL}^G} \right) = - \gamma _L\left( {\Omega _L^{\kappa {\prime}} - \Omega _L^G} \right) - \rho Va_f\left( {z_{c\kappa {\prime}} - z_{cG}} \right)$$

In the case of a free fall, both terms at the left side of Eq. (38) must give negative values because the drop must release energy to deform its shape, i.e. it must do work. By the first term, the acceleration and the difference of center of mass are both positive, resulting in a negative term due to the minus sign ahead of it. The second term is also negative, because the minus sign, the negative value of the areas difference, receding wetting, and the value of *τ*, which is negative due to the following reason: during the de-wetting of the drop contour, the tension *τ* is the force that the liquid must overcome to recede on the solid surface, i.e., to “create” solid surface. This surface tension is the opposite of the previously defined surface energy of the solid *γ*_S_, given as

40 $$\tau = - \gamma _S$$

Under this consideration, Eq. (39) can be written as

41 $$\gamma _{SL} - 2\gamma _S = \frac{{ - \rho {\kern 1pt} Va_f\left( {z_{c\kappa \prime } - z_{cG}} \right) - \gamma _L\left( {{\mathrm{\Omega }}_L^{\kappa \prime } - {\mathrm{\Omega }}_L^G} \right)}}{{{\mathrm{\Omega }}_{SL}^{\kappa \prime } - {\mathrm{\Omega }}_{SL}^G}},\quad a_f = + g_E,\;free\,fall$$

Summarizing, in the case of the free fall, *a*_2_ = −*g*_*E*_ (capsule is falling) and *a*_1_ = −*g*_*E*_ (terrestrial acceleration acting on the droplet in rest). So, the net acceleration that deforms the droplet for a very short time during the free fall is *a*_*f*_ = +*g*_*E*_, i.e., a short duration deformation force from bottom to top. However, once the drop reaches the equilibrium inside the capsule, it will be found within a system with zero acceleration, i.e., a* = 0 (weightless). In consequence, at the end of the experiment (configuration *κ*′), the drop will be resting in a state very close to the stable equilibrium inside an inertial system.

The configuration *κ*, as mentioned before, is reached ideally after the end of a very fast wetting process that started spontaneously with the configuration *o* without the effect of the gravity. In the present method, however, the configuration *κ* is calculated in a special way: the capsule with a drop in the configuration *G*, i.e., resting under the effects of the terrestrial gravity (*a*_1_ = −*g*_*E*_ *, g*_*E*_ = 9.81 m/s^2^) is moved downward with *a*_2_ = −*g*_*E*_, (free fall). This results, as seen above, in a sudden upward acceleration *a*_f_ = +*g*_*E*_ that deforms the drop for some moment (milliseconds in practice) until it reaches the weightlessness state (a* = 0) inside the capsule (inertial system), the so-called configuration *κ*′. The configuration *κ* is the result of an idealized advancing wetting process that results in stable equilibrium, while the configuration *κ*′ is the end of a de-wetting process that, under experimental conditions, ends in a metastable equilibrium very close to the stable equilibrium. The present model proposes that the difference between *κ* and *κ*′ is negligible. This assumption is used by the model to solve the system given by Eqs. (25) and (41).

By analyzing the images provided by the high-speed camera using Axisymmetric Drop Shape Analysis (ADSA) or ellipse matching, it is possible to find the frames where the droplet is resting free of vibrations with a perfectly spherical shape (the ellipse matching is highly recommended). These images correspond to the configuration *κ* ≈ *κ*′ of the model. The values of the interfacial areas $\Omega _L^{\mathrm{o}}$, $\Omega _L^\kappa$, and $\Omega _{SL}^\kappa$ can be obtained by the drop volume, the matching method and the formulas that are presented below, to obtain the value of the parameter *κ*, using the Eq. (24).

It is important to mention, that for the application of any drop-shape analysis method it is essential that the drop is completely axisymmetric. In other words, the wetting has to be completely isotropic. Otherwise it would be necessary to analyze the geometry of the drop in three dimensions. Some manufacturers of drop shape analyzers, like Krüss GmbH (Germany), have already begun to develop top-view distance optical methods for capturing the curvature of the drop, in order to measure contact angles on curved solid surfaces and in depressions which are not easily accessible to the classical drop shape analysis. The combination of this technology with the method presented, could make possible its application for non-axisymmetric drops on anisotropic surfaces.

The values of the interfacial areas $\Omega _L^G$, and $\Omega _{SL}^G$ can be obtained from the first frames some milliseconds before starting the free fall. This instant corresponds to the drop resting in equilibrium under the effects of gravity, the configuration *G*. The drop centers of mass *z*_*CG*_ and *z*_*Cκ*′_ can be calculated from the initial images and the images of configuration *κ* ≈ *κ*′, by applying the Eq. (51) given below. The exact value of the acceleration *a*_*f*_, can be obtained from the accelerometer of the instrument (see the Experimental Section).

The values of *γ*_S_ and *γ*_SL_ can be obtained by solving the system given by the Eqs. (25) and (41). For the application of this method, it is only necessary to know the value of the surface tension of the liquid, its density, and the drop volume.

The precision of this method is highly dependent on the precision of the interfacial areas measurement. During the energy release of the drop, it could move some micrometers on the camera view-axis. As a consequence, the scale of the frames could change a little bit during the process. For this reason, the drop shape matching algorithms must be carefully scaled to evaluate the drop shapes of the configurations *κ* ≈ *κ*′ and *G* with the same drop volume.

The artificial accelerations produced inside the capsule result in drop deformations given by *a*_*f*_. Two cases of spheroidal caps (http://keisan.casio.com/exec/system/1223382199; http://keisan.casio.com/exec/system/13581717527)^40,41^ must be differentiated to calculate the values of the solid–liquid Ω_SL_ and the liquid–air Ω_L_ interfacial areas: a* = 0 and a* < 0.

Case I: sphere cap (http://keisan.casio.com/exec/system/1223382199), a* = 0

The radius of the solid–liquid interface is given by

42 $$R = \sqrt {h\left( {2b - h} \right)}$$

where *h* is the height and *b* the radius of the partial sphere, according to Fig. 2b.

The volume of the partial sphere is given by

43 $$V = \frac{\pi }{6}h\left( {3{\kern 1pt} R^2 + h^2} \right)$$

The surface area, the interfacial area liquid–gas is given by

44 $${\mathrm{\Omega }}_L = \pi \left( {R^2 + h^2} \right) = 2{\kern 1pt} \pi {\kern 1pt} b{\kern 1pt} h$$

And the interfacial area solid–liquid is given by

45 $${\mathrm{\Omega }}_{SL} = \pi {\kern 1pt} R^2$$

Case II: oblate cap,^40^ a* < 0

The radius of the solid–liquid interface is given by

46 $$R = b{\kern 1pt} \sqrt {1 - \left( {1 - \frac{h}{c}} \right)^2}$$

The volume of the oblate is given by

47 $$V = \frac{\pi }{3}{\kern 1pt} b^2{\kern 1pt} c\left( {\frac{h}{c}} \right)^2{\kern 1pt} \left( {3 - \frac{h}{c}} \right)$$

where *b*, *c* and *h* are the magnitudes according to Fig. 2b.

The interfacial area solid–liquid is given by

48 $${\mathrm{\Omega }}_{SL} = \pi {\kern 1pt} b^2\left[ {1 - \left( {1 - \frac{h}{c}} \right)^2} \right]$$

And the surface area—the interfacial area liquid–gas—is given by

49 $${\mathrm{\Omega }}_L = \pi {\kern 1pt} b{\kern 1pt} c{\kern 1pt} \left\{ {{\textstyle{b \over c}} - \left( {1 - {\textstyle{h \over c}}} \right)\,\left[ {1 + \varepsilon ^2{\kern 1pt} \left( {1 - {\textstyle{h \over c}}} \right)^2} \right]^{1/2} + {\textstyle{1 \over \varepsilon }}{\kern 1pt} \left( {ar\,{\mathrm{sinh}}{\kern 1pt} \left( \varepsilon \right) - ar{\kern 1pt} {\mathrm{sinh}}{\kern 1pt} \left[ {\varepsilon {\kern 1pt} \left( {1 - {\textstyle{h \over c}}} \right)} \right]} \right)} \right\}$$

where:

50 $$\varepsilon ^2 = \frac{{b^2}}{{c^2}} - 1 \ge 0$$

In the cases of both sphere and oblate cap, the center of mass of the cap can be calculated using^40^

51 $$z_c = \frac{h}{4}\frac{{4 - \frac{h}{c}}}{{3 - \frac{h}{c}}}$$

## Application of the Method

### Apparatus

The instrument constructed for the purpose of this study consists of a vertical tower three meters high with all the necessary elements to control the accelerated movement of a capsule containing the solid–liquid–gas system during a time span from 600 to 750 ms.

The tower (Fig. 2c) was originally constructed to allow the motion of the capsule under different acceleration values upwards and downwards. However, for the purpose of the present paper, it was used only in free-fall modus, i.e., as a microgravity tower.

The components of the instrument are as follows: the upper ignition device (1), the lower ignition device (2), the displacement tower (3), the acceleration device (4), the braking or damping mechanism (5), the liquid dosing device (6), and the capsule with the sample (7). Further, the instrument is connected to a computer with the software necessary to control the devices and evaluate the data.

The upper ignition device (1), as well as the lower ignition device (2), are electromagnetic mechanisms designed to release the anchored capsule so it can move freely by the action of the acceleration device. They are designed to minimize the vibrations during the ignition. The displacement tower (3) is designed to guide the capsule in the vertical direction without vibration and minimizing the effects of friction. The acceleration device (4) actuates the capsule in values of constant acceleration using the linear increase of the speed starting from the rest (zero velocity). This device is capable of moving the capsule both upwards and downwards. In the case of the downward drive with the acceleration of *–g*_*E*_ (Earth gravitational field), it is possible to let the capsule simply fall free with the help of the guide elements of the shift tower. The acceleration device may be a servo motor capable of producing constant accelerations upwards or downwards of any value or a mechanical device which, based on a combination of pulleys, moves the capsule in discrete acceleration values.

The damping mechanism (5) consists of a mechanical, pneumatic or hydraulic arrangement capable of decreasing the velocity without increasing the acceleration above 20*g*_*E*_ to not damage the devices inside the measuring capsule. Depending on the orientation of the movement, it can be installed on the top or bottom of the tower.

The liquid dosing device (6) must be able to dose small droplets from 5 µL to 100 µL on the sample surface inside the capsule. It can consist of an arrangement of a micro-pump and dosing cannula or a mechanic or electronic micropipette. In the first case, it could be attached and moved together with the microcapsule. In the case of a micropipette, it could be triggered independently of the tower structure.

The measuring capsule consists of a high-speed camera (100–1000 frames per second, fps), a sample stage (XYZ), a diffuse light source, an accelerometer, a vibrometer and a refrigeration unit (ventilator) (Fig. 2d). This device can obtain images or video of the drop during its motion with good resolution at higher fps values. The diffuse light must provide a good illumination and allows a good contrast to obtain sharp drop contours. The stage will be used to put the sample in the optimal position for the experiment. The accelerometer and the vibrometer measure the values of the acceleration (Z-Axis) inside the capsule and the values of the accelerations in X- and Y-axis to evaluate the vibration during the experiment

### Materials

The set up of the method was realized with polypropylene (PP) and polytetrafluoroethylene (PTFE) surfaces.

The PP samples were manufactured by injection of HD1200MO (Borealis AG, Austria) in a mold of 125 × 12 × 1.5 mm^3^, using a melt temperature of 250 °C, holding the pressure of 300 bars and a mold temperature of 30 °C. The most relevant properties of the homopolymer are listed in Table 1.

**Table 1:** Physical properties of the PP

Property	Value
Density	908 kg/m^3^
Melt flow rate (230 °C/2.16 kg)	8 g/10 min
Tensile modulus (1 mm/min)	1500 MPa
Tensile strain at yield (50 mm/min)	9%
Tensile stress at yield (50 mm/min)	33.4 MPa
Heat deflection temperature (0.45 N/mm^2^)	88 °C
Charpy impact strength, notched (23 °C)	4 kJ/m^2^
Hardness, Rockwell (R-scale)	98

The PTFE samples were high-pure, 0.5 mm thickness films produced by Dyneon 3M (USA). The most relevant properties, according to ASTM D 4894, ASTM D 621, ASTM D 638, and ASTM D 149–95a, are listed in Table 2.

**Table 2:** Physical properties of the PTFE

Property	Value
Bulk density	420 g/l
Specific gravity	2.165 g/cm^3^
Shrinkage	5.7%
Tensile strength	4000
Elongation break	350
Deformation under load, 2175 psi, 24 h	8%
Tensile modulus	94250 psi
Dielectric strength	3.7 kV/ml

The topographic characterization was carried out using high-resolution ScanDisk Confocal Microscopy (SDCM). The SDCM device was a µsurf explorer (Nanofocus AG, Germany). Three different optical objectives were used such as 20X, 60X, and 100X, which provide measure lengths of 800, 260, and 160 µm respectively. The lateral resolutions associated with the three objectives were 1.5, 0.5, and 0.3 µm respectively. Five measurements were carried out on different positions of the samples.

The topographic parameters arithmetic mean roughness *Sa* and developed surface area ratio *Sdr* (Table 3) show that the roughness of both materials is small enough to consider the surfaces smooth for the purpose of the experiments.

**Table 3:** Topographic parameters of the surfaces

	Length scale					
	PP			PTFE		
Parameter	20X	60X	100X	20X	60X	100X
*Sa*, nm^a^	105	64	101	636	955	815
*Sdr*, %^b^	0.036	0.094	0.223	0.662	2.447	4.380

^a^ Arithmetic mean roughness

^b^ Developed surface area ratio

## Results and Discussion

**Fig. 3:**
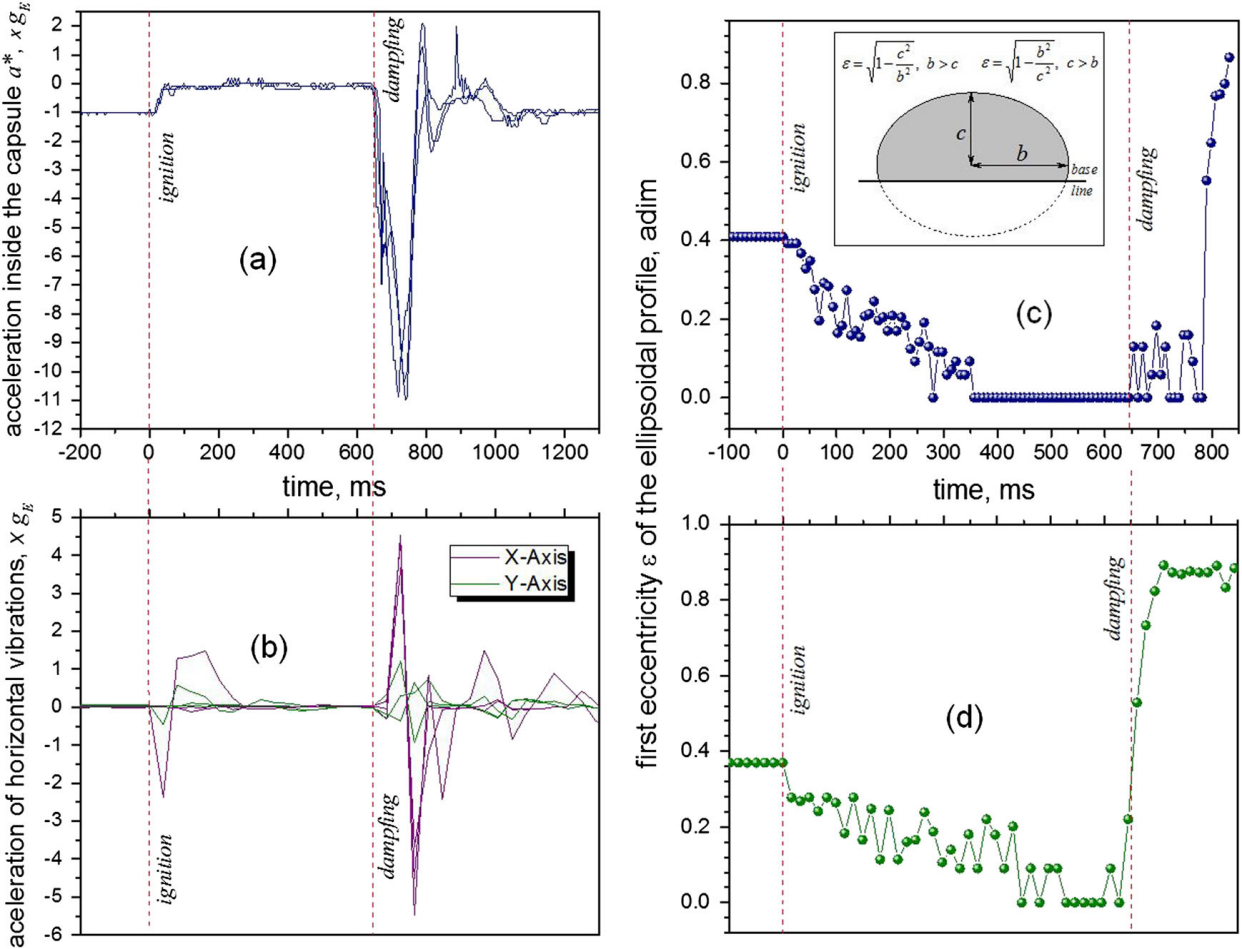
Wetting dynamics during the release of gravity. **a** The acceleration inside the capsule during three experiments of free fall was measured during a trajectory of 2 m. The experiment using the prototype allows a frame of about 600 ms of microgravity. **b** The vibration was quantified by means of the accelerations produced in X- and Y- axis using the USB Vibration Logger DL-131G. These vibrations are important during the first milliseconds of the drop and transmit mechanical energy to the drop. **c** Vibration release of water drops on a PP surface: the drop shape eccentricity for 76 frames were analyzed from the 480 available (750 fps video recording). **d** Vibration release of water drops on a PTFE surface: The drop shape eccentricity for 38 frames were analyzed from the 480 available (750 fps video recording)

Before performing the experiments to determine the parameter *κ*′, the values of the accelerations *a*_2_, *a*_*f*_ and a* were measured using the *R-DAS Tiny rocket accelerometer V4.0* (AED Electronics, USA) installed inside the capsule. The vertical trajectory of the capsule during the free-fall experiment was 2 m, allowing a time lapse of 600 ms of microgravity. The results of three measurements of the acceleration change are presented graphically in Fig. 3a. According to the data, the vertical acceleration of the capsule *a*_2_, was −0.9116 *g*_*E*_, allowing an acceleration for the energy release of *a*_*f*_ = +0.9116 *g*_*E*_. The resulting reduced gravity^42^ inside the capsule was a* = +*0.0884 g*_*E*_ (see Fig. 3a). This value could not be further reduced using the prototype due to the friction of the capsule with the mechanical guides of nylon filaments installed to maintain the horizontal line during the fall, i.e., to avoid unwanted inclinations due to unbalance. A factor that must also be considered is the aerodynamic, i.e., air resistance since the capsule has an aerodynamic front area of 612 cm^2^.

The effect of gravity on the macroscopic advancing contact angle was studied in 2005 by Ababneh et al.^21^ using experiments in a specially modified FALCON 20 business jet (NRC Flight Research Laboratory, Canada) that flies a parabolic arc to produce short periods of reduced net acceleration force. The authors reported important irreproducibility of the contact angle due to the airplane vibration. The use of drop towers also presents the difficulty of the vibration produced during the ignition, i.e., during the release of the capsule to the free fall. Recent experiments realized in 2012 by Diana et al.^22^ using the Drop Tower Facility of the Queensland University of Technology (Queensland, Australia) and Zhu et al.^23^ using the Drop Tower of Beijing (National Microgravity Laboratory, Beijing, China) showed that the simple wire cutting release mechanism that initiates the drop of the experimental rack induces vibrations on the drop. According to ref. 22 the sudden variation of the gravity force induces oscillations in the sessile drop. Indeed, at the beginning of the test, the energy stored in the thin wire that was holding the experimental package and drag shield was released resulting in vibrations in the sessile drop.

To avoid, or at least to reduce the effect of such vibrations, the prototype of the present study was built with an electromagnetic release mechanism. During the release, however, some lateral vibrations (X-Axis, Y-Axis) can be produced as shown in Fig. 3b, where the results of a couple of experiments are graphically presented. These vibrations, measured using a USB *Vibration Logger DL-131G* (Voltcraft, Germany), are due principally to the small torque that the USB-cable of the high-speed camera produced during the release. This fact justifies the use of a wireless system for future experiments.

As Fig. 3b shows, the vibrations are important during the first 300 ms of the experiment. From 500 ms onwards they are negligible.

The experiments using the Drop Tower of Beijing^23^ report a stabilization time from 0.8 to 1.2 s in the case of sessile drops, while by the experiments using the Drop Tower Facility of Queensland,^22^ the vibration time for sessile drops to reach equilibrium is less than 0.5 s. These authors carefully observed the transition from formal to reduced gravity using a 30 fps camera.

It is important to mention that the study of Diana et al.^22^ was oriented to present an initial work to develop a database of contact angles of sessile drops in reduced gravity and to study the dimensional variations due to the increase of the drop size regarding Bond number. The study of Zhu et al.^23^ was oriented to investigate the influence of Bond number on behaviors of liquid drops deposited onto solid substrates and to probe the effectiveness of a simulation model. None of these studies was oriented to study or measure the surface energies of the substrates.

Small drop towers only exist for educational and demonstrative purposes (https://er.jsc.nasa.gov/seh/microgravity_drops.pdf; http://www.dlr.de/schoollab/en/desktopdefault.aspx/tabid-7474/12599_read-31090/). However, there are currently no reports about the study of wetting and surface energies in those devices.

To quantify the stabilization time of the drops during the experiment, the first eccentricity, *ε*, was used. This parameter was defined in Eq. 50 for the oblate (*b* > *c*) case. A perfect sphere (*b* = *c*) with *ε* = 0 in the microgravity region confirms that the effects of vibration were released. According to Fig. 3c, the eccentricity for doubled distilled water drops of 20 µL on the PP surface shows that the vibration is completely released after 350 ms of the ignition.

In the case of the drop of PTFE (Fig. 3d), the vibrations on 20 µL drops are completely released just starting from 570 ms. The hydrophobicity of this surface makes the stabilization period longer.

The images corresponding to the last frames before the damping (500–595 ms) were analyzed for the sessile water droplets of 5, 10, 15, 20, 40 and 60 µL on PP and PTFE surfaces. In all the cases, the drops presented a perfect spherical shape. The calculation of the interfacial areas was possible using Eqs. (44) and (45). The values obtained for the parameter *κ*′ on both surfaces are listed in Table 4. Five measurements corresponding to five different drops were made for each volume on each surface at 22 ± 1 °C of temperature and 60 ± 2% of relative humidity. The dependence on the drop volume will be discussed later.

**Table 4:** Calculated parameter *κ*′ (Eq. (24))

Drop volume (µL)	PP		PTFE	
5	0.57525	±0.02064	0.67899	±0.02620
10	0.56863	±0.00697	0.61133	±0.02046
15	0.53793	±0.00231	0.67426	±0.02353
20	0.54818	±0.01703	0.64825	±0.02307
40	0.53651	±0.02088	0.59016	±0.01545
60	0.53611	±0.03623	0.59055	±0.02714

**Fig. 4:**
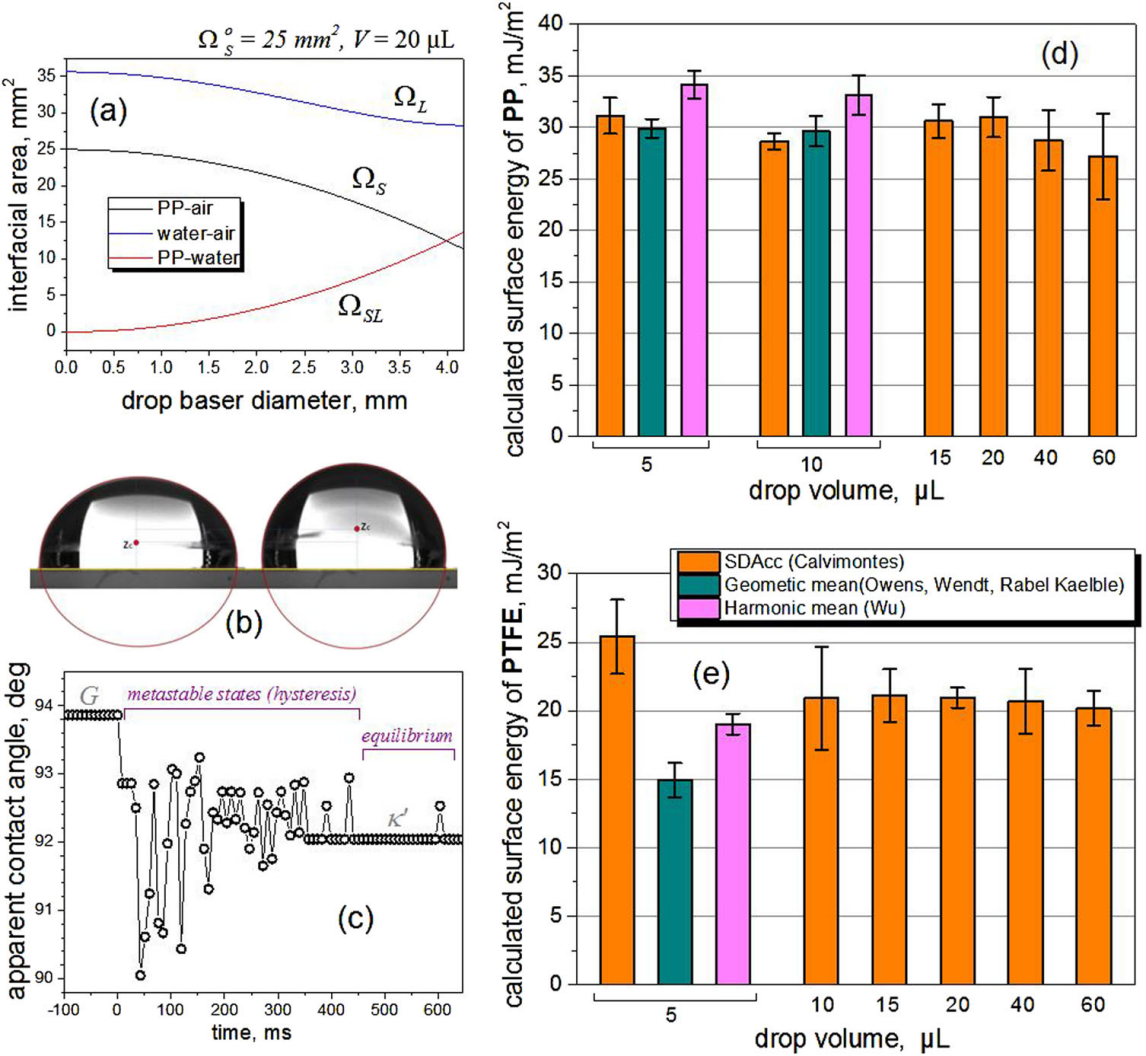
Interfacial changes and calculated surface energy. **a** Change of interfacial areas from the hypothetical configuration *o* to the configuration *κ*′, by assuming *κ* = *κ*′. (**b**) Drop shape differences between configurations *G* and *κ*′. Left: profile of a sessile 40 µL water drop on a PP surface in the *G* configuration (gravity). Right: the same drop in the absence of gravity in the configuration *κ*′. **c** Contact angle hysteresis of a 20 µL water droplet on a PP surface. Transition from stable state G, followed by metastable states (energy release) toward the equilibrium state of the configuration *κ*′. **d** Comparison between the calculated values of *γ*_S_ for the system PP–water–air obtained by the methods Owens et al., Wu, and Sessile Drop Accelerometry (SDAcc). The error bars represent the standard deviation of the mean. **e** Comparison of the calculated values for *γ*_S_ for the system PTFE–water–air obtained by Owens, Wu, and SDAcc. The error bars represent the standard deviation of the mean

The changes of the interfacial areas from the hypothetical *o*-configuration to the configuration *κ* *≈* *κ*′ are presented in Fig. 4a for the special case of a 20 µL water droplet on a PP surface. According to this, the interfacial water–air area decreases during the ideal wetting from the configuration *o* to *κ*′ until reaching a minimum in the equilibrium.

Before initiating the free fall, the interfacial areas at the *G* configuration, i.e., the sessile drop under the effects of the gravity, were measured using the drop images. The drop mass centers in the *G* and *κ*′ configurations were also measured for each experiment. Figure 4b shows an example of a 40 µL water drop on a PP surface.

During the free fall, the water drop experienced receding wetting during approximately 450 ms in case of the PP surface. During this period, the system went through multiple metastable states that can be observed by the contact angle hysteresis in Fig. 4c. The contact angles decrease from 93.8° to 92° due to the release of gravity. The oscillations due to the energy barriers of the surface combined by the mechanical microvibrations due to the electromagnetic release lead the drop to the equilibrium (*κ*′) through multiple metastable states.

The interfacial energies were calculated by solving the system given by Eqs. (25) and (41) for each experiment, i.e., for each pair of images corresponding to the *G* and *κ*′ configurations.

To compare the proposed method, which in the future will be called Sessile Drop Accelerometry (SDAcc), with the Owens-Wendt^28^ and Wu^30^ methods, measurements with a Mobile Surface Analyzer –MSA (Krüss GmbH, Germany) were realized using doubled distilled water and diiodomethane (Sigma-Aldrich). For the PP surfaces, two drop sizes were used: 5 and 10 µL. The results are presented in Fig. 4d.

In the case of the PTFE surfaces, the measurements using the MSA instrument were only possible using 5 µL because larger drops bounce upon touching the surface due to the dosing mechanism. The results are presented in Fig. 4e.

**Fig. 5:**
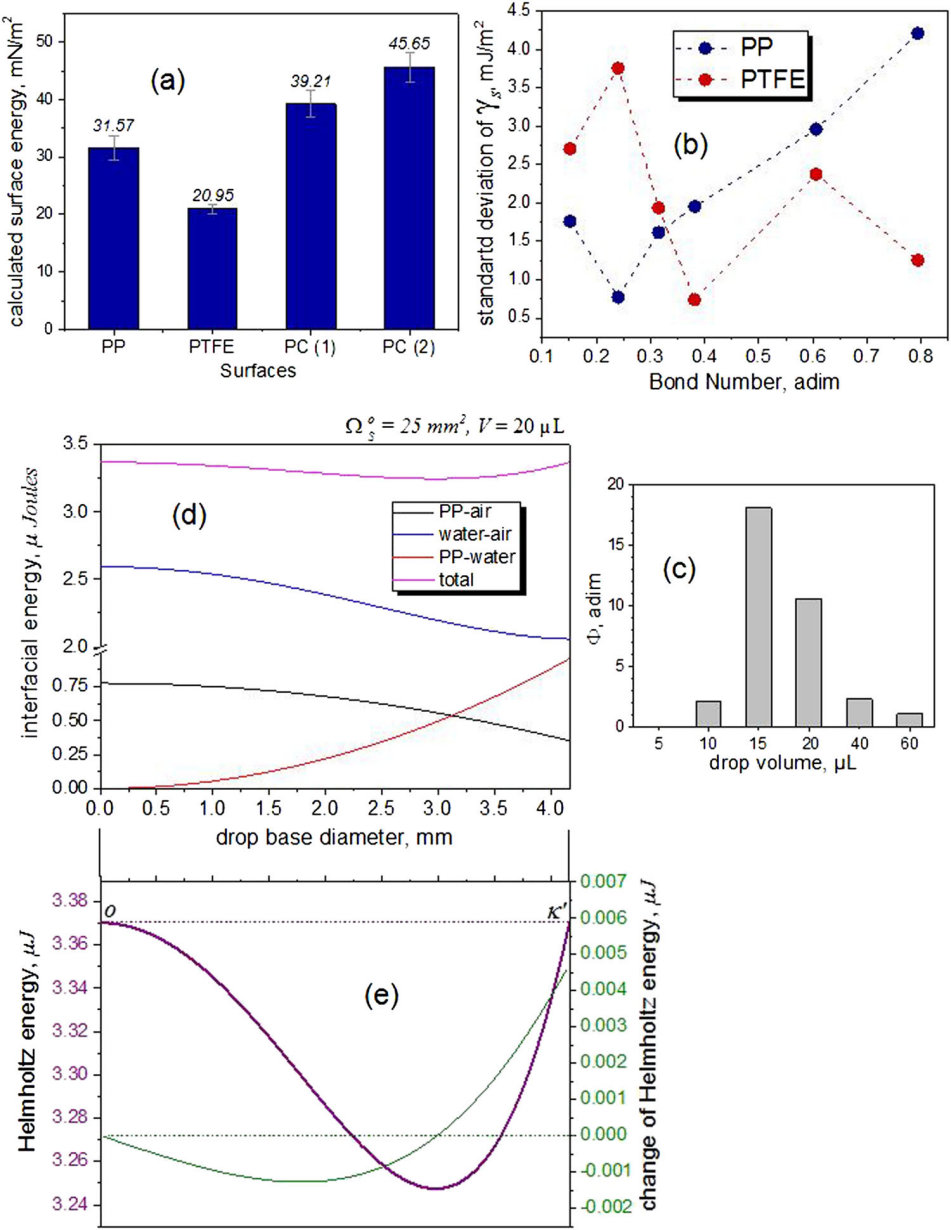
Additional results, drop volume optimization and simulation of wetting without gravity. **a** The method was also used to measure the surface energy of a piece of Makrolon ® Polycarbonate (PC) (sides 1 and 2). According to the average of various references reported by Accu Dyne Tests™ (https://www.accudynetest.com/polytable_03.html?sortby=contact_angle): PP 30.21 ± 2.88 mJ/m^2^ (37 references); PTFE 21.41 ± 3.27 mJ/m^2^ (44 refs.); PC 42.68 ± 4.78 mJ/m^2^ (9 refs.). **b** Correlation between measurement accuracy and drop size for the PP-system and PTFE-system using the Bond (Eötvös) number. **c** The energy ratio Φ of the gravitational potential and the work done by the liquid–air interface could be used to find the optimal droplet volume. **d** The energies of the water–air and PP–air interfaces spontaneously do work to build the PP–water interface. **e** Using the values of the energies in the configuration κ′ it is possible to reconstruct the idealized spontaneous wetting of a drop in the absence of the gravity (see Section Theoretical Fundamentals of the Method)

Additionally, SDAcc was applied to measure the surface energies of both sides of a Makrolon® Polycarbonate plate (Covestro AG, Germany). The surface energies measured with drops of 20 µL water are presented in Fig. 5a.

Once the system of the Eqs. (25) and (41) has been solved, it is possible to find all the variables of the experiment, including the apparent contact angles, *θ*_*κ*′_ and *θ*_*G*_, under the effects of the microgravity and at the gravity, respectively. Table 5 presents the variables for the PP and PTFE using doubled-distilled water drops of 20 µL and an initial solid surface of 25 mm^2^.

**Table 5:** Wetting parameters for 20 µL sessile water drops on PP and PTFE

Parameter		PP	PTFE
*γ*_S_	mJ/m^2^	31.0481	20.9384
*γ*_SL_	mJ/m^2^	70.2298	64.9132
*κ*	—	0.54818	0.64825
$\Omega _S^{\kappa {\prime}}$	mm^2^	11.3585	13.1434
$\Omega _S^G$	mm^2^	10.3006	12.7835
$\Omega _L^{\kappa {\prime}}$	mm^2^	28.2898	28.4698
$\Omega _L^G$	mm^2^	28.3079	28.4293
$\Omega _{SL}^{\kappa {\prime}}$	mm^2^	13.6414	11.8566
$\Omega _{SL}^G$	mm^2^	14.6993	12.2164
*γ*_L_ $\Omega _L^\kappa$	µJ	2.0595	2.0726
*γ*_L_ $\Omega _L^G$	µJ	2.0608	2.0690
*γ*_S_ $\Omega _S^\kappa$	µJ	0.3527	0.2752
*γ*_S_ $\Omega _S^G$	µJ	0.3198	0.2677
*γ*_SL_ $\Omega _{SL}^{\kappa {\prime}}$	µJ	0.9580	0.7696
*γ*_SL_ $\Omega _{SL}^G$	µJ	1.0323	0.7930
*z*_*c*_ *κ*_′_	mm	0.8146	0.8885
*z*_*cG*_	mm	0.7596	0.8552
*Aκ*′	µJ	3.3702	3.1174
*A*_*G*_	µJ	3.4130	3.1303
*W*_*M*_ *=* *A*_*G*_−*A*_*κ*′_	µJ	0.0428	0.0129
*θκ*′	°	92.04	99.62
*θ*_*G*_	°	90.86	100.99

After “switching off” gravity, the apparent liquid contact angles on hydrophobic surfaces (*θ* > 90°) tend to decrease, according to previous observations of Ababneh et al.,^21^ who studied the contact angles of water on Teflon-coated silicon wafers during the reduced gravity period of parabolic arc flights. In those cases, the gravity is initially pressing the ellipsoidal (oblate) profile of the droplet on the surface and increasing its contact angle. Once the gravity is reduced, the droplet rises a little and the contact angle of its ellipsoidal profile with the surface decreases. In the experiment of Ababneh et al. the decrease was from 126° to 121°. This observation agrees with the results of Table 5 for the PTFE surface (decrease from 101° to 99.6°). However, on hydrophilic surfaces (*θ* < 90°) the effect of “switching off” gravity should, in principle, be the reverse: if the contact angle is smaller than 90°, the upward movement of the droplet profile due to its change of center of mass and the de-wetting will increase the contact angle. According to this, the behavior of the droplet on the PP surface (see Table 5) corresponded to a hydrophilic system, i.e., a slight increase of contact angle due to the release of gravity. This could be explained by the fact that this system is at the hydrophobic–hydrophilic boundary due to its contact angle very close to 90°. However, according to the experimental results, the standard deviations of the measurements for *θ*_*κ*′_ and *θ*_*G*_ were only 1.92° and 2.05° respectively. Another interpretation for this behavior is that this is an effect of the receding wetting due to the gravity release: the effect of micro- and nano-roughness of the surfaces by the stick-slip effect would also be modifying—decreasing—the contact angles on the contour line of the drop at the configuration *κ*′. This interpretation, however, does not correlate with the fact that the drops had a perfect spherical dome shape in the configuration *κ*′. To better interpret these results, the application of the method on different hydrophilic surfaces will be carried out in the next future.

According to Eq. (24), *κ* is a parameter independent on the drop size. However, this property is only valid if the surface tension of the liquid and the gravity forces are properly balanced. Indeed, there are two forces that principally produce the drop shape:^22^ the surface tension of the liquid, which tends to minimize the area of the surface (producing the spherical shape) and the gravitational force which tends to flatten the drop. The competition between both effects yields the equilibrium shape under the effect of the gravity. If the drop volume is too small, the surface tension will dominate, and the effect of the gravity will be negligible making impossible or very difficult to construct Eq. (41) using SDAcc. In those cases, the drop shape analysis could also lose accuracy if the image resolution of the instrument is not the best. On the contrary, if the drop is very large, the gravity will dominate, making the drop shape more and more dependent on the mass and viscosity than on the surface tension. In these cases, the inertia of the drop will additionally produce longer vibrations during the energy release making the measurement accuracy technically impossible. Therefore it is necessary to find an optimal drop volume range for the evaluation of the interfacial energies using SDAcc.

The Bond number (*Bo*) also called Eötvös number (*Eö*),^43^ is a dimensionless number that was used recently to evaluate the effect of microgravity on drops.^22,23^ *Bo* gives the ratio between the gravity force and the surface tension force

60 $$Bo = E {\"o} = \frac{\Delta \rho {R^{2}}g} {\gamma_{L}}$$

where Δ*ρ* is the density difference between the liquid and the vapor, R is the wetting radius, *g* is the gravity, and *γ*_L_ the fluid surface tension.

Figure 5b correlates the repeatability of the measurements with the dimensions of the drops expressed as the Bond number. According to the data, the best drop volume to evaluate the PP-system is 10 µL, while 20 µL is the best volume to evaluate the PTFE-system.

An alternative method to find the optimal drop volume for a given solid–liquid–air system, could be by comparing the gravitational potential energy (see Eq. (41)) with the work made by the liquid–air interface during the de-wetting process by means of the ratio Φ, defined as

61 $${\mathrm{\Phi }} = \left| {\frac{{ - \rho {\kern 1pt} Va_f{\kern 1pt} \left( {z_{c\kappa \prime } - z_{cG}} \right)}}{{\gamma _L{\kern 1pt} \left( {{\mathrm{\Omega }}_L^{\kappa \prime } - \Omega _L^G} \right)}}} \right|$$

Applying this criterion to the PP system, we have that this parameter reaches a maximum value for 15 µL, according to Fig. 5c. This means that at this drop volume the release of the gravitational energy is large enough to produce both, the deformation of the droplet shape and the receding wetting on the solid-liquid interface, in order to form a near perfect spherical dome. To apply this method, however, it is anyway necessary to carry out some measurements at different drop volumes.

If *κ* = *κ*′, the values obtained by the measurements can be used to construct quantitatively the curves showed schematically in Fig. 1c, 1d. The idealized spontaneous wetting in the absence of gravity is possible by the decrease of the Helmholtz energy of the system due to the transformation of the water-air interface into the solid-water interface. The solid–air interface also contributes to building the solid–liquid interface (Fig. 5d).

The total energy of the interfaces decreases up to reach a minimum (see Fig. 5e). However, some part of the energy has been stored as internal energy into the liquid. This energy will complete the spontaneous wetting up to reach the configuration *κ*.

**Fig. 6:**
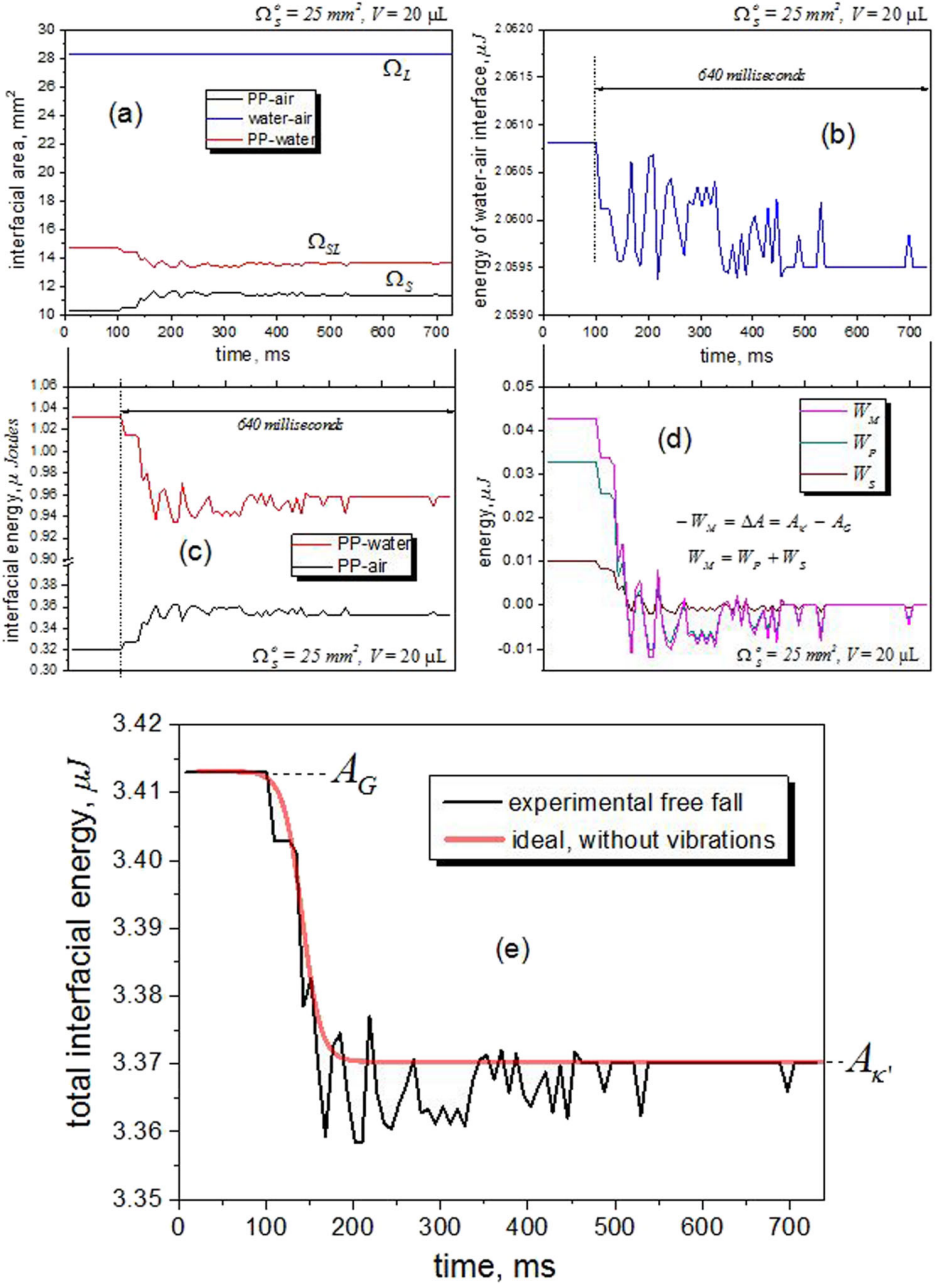
Thermodynamics of de-wetting during the gravity “switch-off”. **a** The energy release of the droplet during the free fall is produced principally by the de-wetting at the solid–liquid interface. **b**, **c** Changes in the interfacial energy water–air are very small in comparison with the changes of the other interfaces. **d** The energy release of the drop during the free fall is equal to the work that the gravitational field was making to flatten the drop before the free fall. **c** The main challenge for the further development of the method is to avoid the vibrations produced by the instrument

The high-speed camera images were used to calculate the changes in the interfacial surfaces. Figure 6a–c show these changes regarding interfacial areas and energy. The water interface presents almost no change during the free fall in comparison with the energy changes in the solid–liquid interface. Indeed, the major part of the energy is released by the solid–liquid interface due to the de-wetting process resulting from the “switching off” of the gravity.

The total interfacial energy released during the free fall of the drop is equal to the work *W*_M_ that the gravitational field was making on the droplet at the configuration *G*. Both components of this work, the mechanical (drop flattening), *W*_P_, and the adhesion work, *W*_S_, are released by the droplet during the free fall (Fig. 6d). In other words, the work *W*_M_ is exactly the difference between the total interfacial energies of the configurations *G* and *κ*′.

Ideally, the energy release during the free fall without vibrations could be represented by a sigmoid, as shown in Fig. 6e. This process is very fast, between 50 and 100 ms. The challenge of the further development of SDAcc is to minimize the vibrations produced by the capsule release to gain measurement accuracy.

## Conclusion

A method, the Sesile Drop Accelerometry (SDAcc), was presented to study and measure the interfacial solid–gas and solid–liquid energies submitting a sessile drop to uniform and controlled microgravity environment using a small acceleration tower designed for the use inside the laboratory.

The mathematical model proposed to calculate the interfacial energies is based on the wetting thermodynamics taking into account the energies of the equilibrium configurations of the liquid drop before and after the release of energy due to the free fall without the need to measure apparent contact angles. The presented model bases on the thermodynamic equilibrium of the interfaces and not on the balance of bi-dimensional tensors on the contour line. For this reason, the model does not follow Young’s equation, as the current surface wetting characterization techniques usually do.

For the application of the method, only one liquid with a known value of surface tension is needed. In the case of using water, the optimal volume of the drops would be between 10 and 20 µL to allow the action of both the surface tension and the gravitational force determine the drop shape. The greatest obstacle to the method accuracy is the vibration produced during the start of the wetting process. However, the experimental results are showing that the release of these vibrations occurs for a very short time. The high-speed camera of the instrument allows obtaining images of the drops for an additional time free of vibrations.

The calculation of surface energies of PP, PTEF, and PC are showing that the values obtained by using the method with double-distilled water agree with the values measured by other methods and with the Literature.

The instrument and its evaluation method open new possibilities to develop surface characterization procedures by submitting the solid–liquid-system to artificial generated and uniform force fields.

Finally, the instrument can also be used to develop a database of contact angles of sessile drops in reduced gravity without the necessity of using space laboratories, parabolic flights or big drop towers.

### Availability of data, materials and methods

All the materials described in the article, including all relevant raw data like video sequences, spreadsheets, algorithms, topographical data, technical details and images of the prototype, are freely available by requesting the author.
